# Bibliography

**Published:** 1898-04

**Authors:** 


ANATOMY, DESCRIPTIVE AND SURGICAL. By Henry Gray, F.R.S. Philadelphia and New York: Lea, Brothers & Co.

In the review of this standard work in this journal, December, 1897, the important fact was overlooked that an entire revision of the illustrations had been made wherever they related to dental anatomy. Heretofore, in books of this character, very little if any care has been exercised to make the dentures absolutely correct. An examination of the illustrations from Figs. 154 to 568, twenty in all, it will be noticed that the teeth have been drawn by one entirely familiar with dental forms, which adds materially to its value from a dental point of view, as well as increasing the value of the book for dental teaching.

THE MONTHLY CYCLOPÆDIA OF PRACTICAL MEDICINE AND UNIVERSAL MEDICAL JOURNAL. Edited by Charles E. de M. Sajous, M.D., Philadelphia. Philadelphia: F. A. Davis Co., Publishers.

"The companion publication of the new annual—the Monthly Cyclopædia of Practical Medicine, the continuation of the Universal Medical Journal—is designed to play a much more important *rôle* than the latter did as an aid to subscribers to the work. It will consist of forty pages of matter intended to portray, in an easily assimilated form, the practical suggestions embodied, first, in the literature of the previous year, and, secondly, in the current literature, the whole making at the end of each year an additional volume of nearly five hundred pages."

This is from the Introduction by the editor of Volume I., new series, and of Volume XII., old series.

The initial number is a very great improvement over the former issue, valuable as that always was considered, and it must commend itself in every respect to medical readers, and in this category should be classed dentists. The number is yearly growing who have neither the time nor the means to read or subscribe for the medical literature of the world. Here we have it condensed and served in an interesting form at very moderate cost.

The first number contains an interesting and valuable article by the editor, on the treatment of cancer by interstitial injections of alcohol. His reasons for the use of this agent, injected hypodermically, with the cases cited to support them, seem to demonstrate that this agent deserves an extended trial.

This Cyclopædia can be most cordially recommended to all who wish to keep in touch with the progress of the healing art.

Proceedings of the National School of Dental Technics. For the Years 1893–96. Published 1897.

This pamphlet of one hundred and one pages is filled with the proceedings of this association at Chicago, Old Point Comfort, Asbury Park, and Saratoga. The subject-matter is chiefly composed of papers and reports. These, on the whole, are interesting reading and give many suggestions worthy of the serious consideration of dental teachers.

The American Dental Weekly. Issued every Thursday. B. H. Catching, D.D.S., Chief Editor. Atlanta, Ga.

Notice of this new dental journal has been deferred for the reason that while the writer has great faith in Dr. Catching's energy and ability, he has been very sceptical in regard to the continuance of a weekly publication and, therefore, preferred to wait for time to remove this doubt, and at the same time enable the reader to form a definite opinion of its value.

Dental weeklies have lived in the professional mind more as a hope than a reality, and until this one was established there was slight reason to expect that any overworked dentist would attempt such a hazardous financial experiment. That Dr. Catching and his co-laborers have been equal to this creates a sense of confidence that it will be continued upon lines laid down. Its short excerpts spiced with some valuable original matter will doubtless meet the demands of many, but it is doubtful whether a weekly of this size can ever become the medium for the original work of the dental profession. This time can alone prove.

The courage of the projectors and their ability for hard work has been demonstrated, and we wish them unlimited success.

Transactions of the Illinois State Dental Society, at the Thirty-third Annual Meeting, held at Peoria, May 11 to 14, 1897. Chicago: The Dental Review, 1897.

This makes a volume of two hundred and sixty-six pages, a large part of which is unusually interesting matter. Several of the articles have a permanent value, and among these may be noted the paper on "The Diffusibility of Coagulants in Dentine." Nothing better has been given the profession on this subject, and, in the view of the writer, practically disposes of this much disputed subject.

